# Effect of post-exercise ingestion of different molecular weight carbohydrate solutions. Part III: Power output during a subsequent resistance training bout

**DOI:** 10.1186/1550-2783-12-S1-P32

**Published:** 2015-09-21

**Authors:** Leighsa E Van Eck, Anthony L Almada, Margaret T Jones, Andrew Jagim, Joel Mitchell, Jonathan M Oliver

**Affiliations:** 1 Department of Kinesiology, Texas Christian University, Fort Worth, TX 76129, USA; 2 Vitargo Global Sciences, LLC, Dana Point, CA 92629, USA; 3 Health and Human Performance Division, George Mason University, Fairfax, VA 22030, USA; 4 Exercise & Sport Science Department, University of Wisconsin - La Crosse, La Crosse, WI 54601, USA

## Background

To maximize power adaptations, resistance training (RT) should be performed at maximal power output. In sports where more than one training bout is necessary in a day, subsequent RT may be limited by muscle glycogen, resulting in lower power output. High molecular weight (HMW) carbohydrate (CHO) solutions have been shown to result in greater glycogen re-synthesis rates, and greater work output during a subsequent cycling time trial compared to a low molecular weight (LMW) CHO solution. However, the effect of a HMW CHO on RT power output following exhaustive exercise is unknown.

## Methods

Sixteen resistance trained men (mean ± SD; 23 ± 3 years; 176.7 ± 9.8 cm; 88.2 ± 8.6 kg; 12.1 ± 5.6% fat) participated in this study. One-repetition maximum (1RM) back squat (153.3 ± 53.6 kg; 1.7 ± 0.2 1RM:body mass), and VO_2 _max (37.4 ± 4.3 ml·kg·min^-1^) were initially assessed in order to prescribe exercise intensities during experimental trials. In a double-blind, placebo-controlled, randomized cross over design consisting of three testing sessions separated by one week, subjects completed a glycogen depleting exercise bout on a cycle ergometer. Immediately post-exercise, subjects ingested a placebo (PLA), or a LMW or HMW CHO solution (10%) providing 1.2 kg· bw^-1 ^CHO, assigned randomly. Two hours post-ingestion, subjects performed 5 sets of 10 repetitions back squat (75% 1RM) "as explosively as possible". If subjects paused for more than 2 seconds or were unable to complete a rep, resistance was lowered by 13.6 kg. Kinematic and kinetic measurements were sampled at 1000 Hz via force plate and two linear position transducers.

## Results

Average power following ingestion did not differ between CHO solutions until Set 4 (p = 0.108) and Set 5 (p = 0.083). Average power collapsed across the latter Sets was greater following ingestion of the HMW solution (Set 4, 1216 ± 97 W; Set 5, 1143 ± 102 W) compared to PLA (Set 4, 1066 ± 80 W: p = 0.037; Set 5, 1019 ± 89 W: p = 0.048), but not compared to ingestion of LMW (Set 4, 1160 ± 79 W: p = 0.355; Set 5, 1131 ± 92 W: p = 0.852). No difference was observed between LMW and PLA (Set 4, p = 0.275; Set 5, p = 0.077). The difference in average power was driven by velocity, as similar trends were observed in Set 4 and 5 (p = 0.100 and p = 0.066, respectively). Average velocity was higher following ingestion of HMW (Set 4, 0.63 ± 0.03 m·s^-1^; Set 5, 0.62 ± 0.03 m·s^-1^) compared to PLA (Set 4, 0.56 ± 0.04 m·s^-1^: p = 0.050; Set 5, 0.56 ± 0.04 m·s^-1^: p = 0.032), but not LMW (Set 4, 0.61 ± 0.03 m·s^-1^; p = 0.422; Set 5, 0.61 ± 0.03 m·s^-1^: p = 0.074), with no difference between LMW and PLA (Set 4, p = 0.220; Set 5, p = 0.769). HMW conferred a likely beneficial effect in Sets 4 and 5 (92.5% and 88.7% likelihood, respectively), compared to PLA; while ingestion of LMW conferred only a possibly beneficial effect (68.7%) and likely beneficial effect (83.9%) in Sets 4 and 5, respectively.

## Conclusions

These data suggest post-exercise ingestion of a HMW CHO solution providing 1.2 kg· bw^-1 ^CHO may allow athletes to sustain power output in a subsequent resistance training session when time between training sessions is limited.

